# Systematic high-resolution assessment of global hydropower potential

**DOI:** 10.1371/journal.pone.0171844

**Published:** 2017-02-08

**Authors:** Olivier A. C. Hoes, Lourens J. J. Meijer, Ruud J. van der Ent, Nick C. van de Giesen

**Affiliations:** 1 Department of Water Management, Faculty of Civil Engineering and Geosciences, Delft University of Technology, Delft, the Netherlands; 2 Witteveen+Bos Raadgevende ingenieurs B.V., Deventer, the Netherlands; 3 Department of Physical Geography, Faculty of Geosciences, Utrecht University, Utrecht, the Netherlands; Pacific Northwest National Laboratory, UNITED STATES

## Abstract

Population growth, increasing energy demand and the depletion of fossil fuel reserves necessitate a search for sustainable alternatives for electricity generation. Hydropower could replace a large part of the contribution of gas and oil to the present energy mix. However, previous high-resolution estimates of hydropower potential have been local, and have yet to be applied on a global scale. This study is the first to formally present a detailed evaluation of the hydropower potential of each location, based on slope and discharge of each river in the world. The gross theoretical hydropower potential is approximately 52 PWh/year divided over 11.8 million locations. This 52 PWh/year is equal to 33% of the annually required energy, while the present energy production by hydropower plants is just 3% of the annually required energy. The results of this study: all potentially interesting locations for hydroelectric power plants, are available online.

## 1 Introduction

Worldwide energy demand stood at 164 PWh/year in 2011, and will be 200 PWh/year in 2020 [1]. With the ongoing depletion of fossil fuels, it is inevitable that alternative energy resources, including hydropower, will have to play an increasingly significant role [2,3]. The common consensus is that renewables will need to furnish more than 50% of the world’s energy consumption within 40 years [4].

Renewable energy sources such as hydropower, biofuels, wind, solar, and geothermal energy currently represent only 15% of the world’s total energy production [1]. However, the contributions from these sources are growing rapidly. Among these sources, hydropower plants currently make the greatest contribution. Although estimates vary, hydropower production in 2012 was estimated at 3.7 PWh and the installed hydropower capacity, was approximately 990 GW– a figure that is growing by an estimated 30 GW per year [5].

Hydropower energy potential is typically divided into a) gross theoretical potential, b) technical potential, and c) economically feasible potential. The gross theoretical potential expresses the total amount of electricity that could potentially be generated if all available water resources were devoted to this use. The technically exploitable potential represents the hydropower capacity that is attractive and readily available with existing technology. The economically feasible potential is that amount of hydropower generating capacity that could be built after conducting a feasibility study on each site at current prices and producing a positive outcome. Technical and economic feasibility strongly varies depending on local conditions, and, therefore, requires in-depth studies at each potential site, which is why we focus on gross theoretical potential.

It has been estimated that there is a global gross theoretical available potential of 36 to 128 PWh/year, a technical potential of approximately 8 to 26 PWh/year, and an economically feasible potential of 8 to 21 PWh/year [6–14]. In ref [9], runoff estimates together with average altitudes by continent were used for the evaluation of gross theoretical hydropower potential by continent. Later, more advanced methods were developed, where modeled discharges and total head differences between coarse grid cells (>50 km scale) were used [12–14], but did not pinpoint individual locations on a fine scale (<1 km) as is the topic of this study.

Hydropower potential can be categorized in terms of pico, micro, mini, small and large hydropower plants. Large hydropower plants are plants with an installed capacity above 10 MW. The potential locations of large plants are generally known. However, the accumulated global potential of small (<10 MW), mini (<1 MW), micro (<0.1 MW) and pico (<0.005 MW) hydropower is in the current practice roughly estimated, at best, and the locations where plants might be installed are generally unknown at global scale.

Recent studies have already made accurate hydropower potential estimations for specific areas [15–17]. Different types of hydrological data and approaches can be used, such as remote sensing and hydrologic modeling, as used for poorly gauged basins [18,19]. Systematic methods applicable at national and regional levels have been generated as well [20–23]. These studies have noted that GIS-based tools combined with hydrological models or data are useful for assessing hydropower for specific areas and could be used on global scale as well. However, the potential and specific locations of, especially micro and mini hydropower, has never been systematically computed globally.

The objective of this paper is (a) to provide a systematic estimate of the global gross theoretical hydropower potential, (b) to provide insight in its distribution between micro, mini, small and large hydropower, and (c) to provide insight in the potential per country and per capita. Moreover, we present an online database in which the exact locations of potential hydropower locations can be found. As a result, our research shows, at a global scale, the varying densities in hydropower potential for, especially, smaller plants; thereby guiding investments at national and regional levels in micro-hydropower programs.

## 2 Methods

The gross capacity of a hydropower plant in a river can be calculated as:

P=ρ⋅g⋅H⋅Q (1)

where *P* is the hydropower capacity (in W), ρ is the density of water (kg/m3), *g* is the gravitational acceleration (m/s^2^), *H* is the head (m) and *Q* is the discharge (m^3^/s). The maximum annual energy production is reached when 100% of the annual runoff is used for hydropower production (i.e. gross potential).

To systematically survey all rivers and their discharge, elevation data were used to delineate a global river network with the average annual discharge for each location or raster cell. We used two standard GIS operations—flow direction and flow accumulation—with the composite runoff data as a weight factor [24]. The Digital Elevation Model (DEM) used in this study was the ‘GMTED2010’ 7.5 arcsec breakline emphasis product. The breakline emphasis product is especially useful for the generation of hydrologic derivatives or distributed hydrologic modeling applications conducted over large areas [25]. The composite runoff data were taken from the UNH-GRDC Composite Runoff Fields V1.0 dataset. This monthly averaged, 30-minute dataset is produced by combining river discharge measurements with a climate-driven water balance model. These composite runoff fields can be regarded as the best global estimate of terrestrial runoff [26].

Only river locations with a drop *H* ≥ 1 meter between two adjacent cells of 7.5 arcsec (≈225m at the equator) and a discharge *Q* ≥ 0.1 m^3^/s were selected as suitable hydropower locations. We were bound to a minimum height of 1 meter by the integer raster data of the GMTED2010 global DEM. For smaller areas, there are elevation maps with a higher level of detail, but these do not allow a consistent global analysis. A combination of *Q* = 0.1 m^3^/s and H = 1 m delivers (with eq 1) the smallest potential hydropower plant locations on our map with a capacity of 1kW or 8,760 kWh per year. In our analysis, we combined the capacity of adjacent potential locations in mountainous regions to prevent cascades of numerous micro-turbines. As a requirement to combine locations, we used the presence of an uninterrupted chain of drops of at least 1 m between river raster cells. Finally, for the categorization over plant size, we used a fixed capacity factor of 0.5. This fixed capacity factor is used when estimating the power production per location since plants never function continuously at 100% due to equipment failures, stoppages for routine maintenance, daily variations in energy demand and seasonal fluctuations in water supply. The actual capacity factor is a design parameter and ranges between 1–99% depending on the purpose of the plant (base or peak load energy supplier), the possibility of building a reservoir, energy price, and the availability of other energy resources.

## 3 Results

### 3.1 Global hydropower potential

In total, global gross theoretical hydropower potential is 52.0 PWh/year divided over 11.8 million locations based on the 7.5 arcsec GMTED2010 elevation data [25] and the runoff data from the Global Runoff Data Centre [26]. This number is approximately one-third of current global energy need [27]. Of course, many of the locations cannot be developed for (current) technical or economic reasons, but the value obtained in this analysis shows the significant potential of hydropower in the future energy mix.

To determine the sensitivity to a minimum height of 1 meter, we also determined the global energy potential for a minimum height difference of 2, 3 or 4 meters between cells in a river. At a minimum height of 2, 3 or 4 meters between two cells, the Global Energy Potential drops to 39.0 PWh / year, 34.0 PWh / year and 29.0 PWh / year, respectively.

Our global estimate is slightly lower than the estimate of ref [12], who estimated about 58 PWh /year, and ref. [14], who estimated 56 to 67 PWh / year depending on the hydrological model used. Our estimate is, however, much lower than the estimate of ref [13], who estimated 128 PWh /year. There are several possible causes to explain the difference in gross hydropower. First, regarding ref [13], it is not clear how they estimated hydropower potential at latitudes above 60 degrees North, as HydroSHEDS currently does not include those data. Second, the authors of refs [12–14] calculated the runoff with global hydrological models, while the GRDC data used by us originates from observed discharges. Third, the hydrological models in refs [12–14] use much coarser grid sizes than those we use (7.5 arcsec), and moreover, we consider water to be in the river network only for discharges above 0.1 m^3^/s. What makes that in our case water particles that fall on hillslopes enter the river network at lower elevations, thus decreasing the potential energy. Compared to these previous studies, our study is the first to provide the exact locations of the potential sites for micro, mini and small and large hydropower plants.

### 3.2 Spatial distribution of hydropower gross theoretical potential

**Fig 1:**
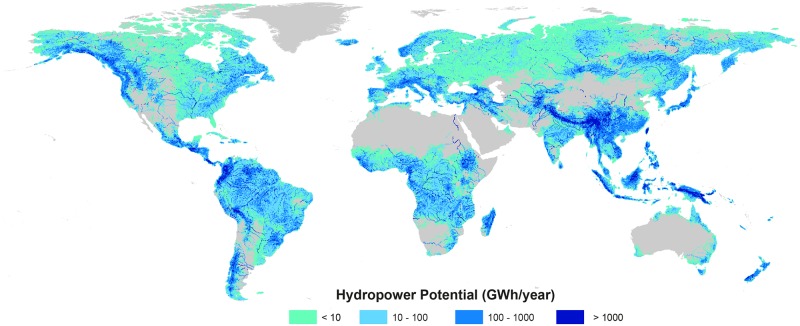
Global map of gross hydropower potential distribution.

**Fig 2:**
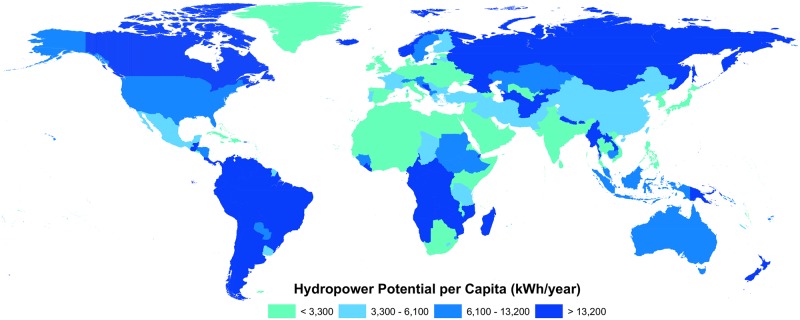
Global map of hydropower per capita per country. The cut-off values correspond to present per capita energy production in China (3300 kWh/year), Europe (6100 kWh/year, and the USA (13,200 kWh/year).

Of all the continents, the greatest contributor is Asia, which represents 48% of the global hydropower potential (Fig 1, Table 1). More interesting are the countries with a high hydropower potential per capita and a low present energy production. For this analysis, we divided the sum of the hydropower potential per country by the population in 2010 [28,29]. We compared the potential production per person with the current average energy output per person in China (3,300 kWh / person / year), the European Union (6,100 kWh / person / year), and the USA (13,200 kWh / person / year) [27]. We found that 46 countries have sufficient potential per capita hydropower energy within their boundaries to cover the needs of the average USA citizen (Fig 2). Furthermore, a total of 71 countries have enough hydropower for an average European’s needs, and 91 countries surpass the present energy need of a Chinese citizen.

**Table 1:** Top 20 countries with the highest hydropower potential per capita.

	Country	Hydropower Potential (TWh/year)	Population in 2010 (x 1,000)	Hydropower potential per capita (KWh/year/person)	Current energy production per capita (KWh/year/person)
1	Bhutan	229	717	319,000	*NA*
2	Iceland	88	318	275,100	52,374
3	Papua New Guinea	1,087	6,859	158,500	*NA*
4	Guyana	109	786	138,400	*NA*
5	Gabon	167	1,556	107,300	907
6	Suriname	54	525	102,800	*NA*
7	Canada	3,064	34,126	89,800	16,473
8	Bolivia	816	10,157	80,400	623
9	New Zealand	275	4,368	62,900	9,399
10	Congo	227	4,112	55,100	*NA*
11	Norway	253	4,891	51,700	23,174
12	Laos	320	6,396	50,100	*NA*
13	Montenegro	29	620	46,100	5,747
14	Peru	1,145	29,263	39,100	1,248
15	Equatorial Guinea	27	696	38900	*NA*
16	Colombia	1,641	46,445	35,300	1,123
17	Zambia	419	13,217	31,700	599
18	Belize	10	309	31,100	*NA*
19	Nepal	789	26,846	29,400	106
20	Chile	494	17,151	28,800	3,568
27	Russia	3,503	143,618	24,400	6,486
37	Brazil	3,630	195,210	18,600	2,438
62	United States	2,564	312,247	8,200	13,246
74	China	7,168	1,359,821	5,300	3,298

Although many countries already make good use of hydropower, we found that several countries with frequent power shortages, such as Bolivia, Zambia, Nepal, Myanmar, and Gabon have extensive high hydropower potential per capita that could increase current production. Some countries have low per capita potential, either because they are flat (e.g. The Netherlands), dry (e.g. Saudi Arabia) or populous (e.g. Bangladesh, Germany, Japan and Nigeria). These countries are likely to rely mainly on other sources to meet their energy needs.

### 3.3 From micro to large hydropower

**Fig 3:**
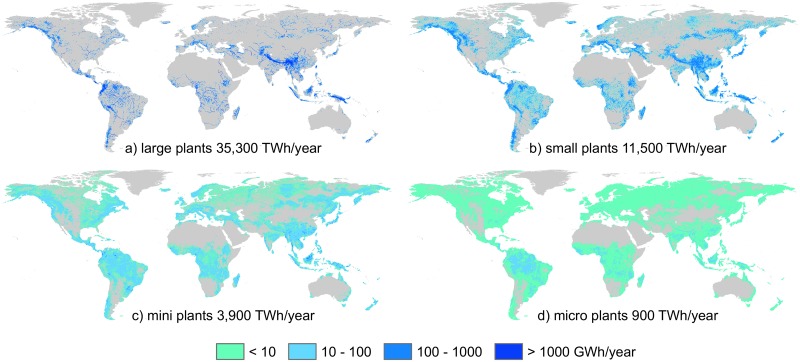
Global number of gross potential hydropower locations of a certain size. Total number of locations found is 1.2 × 10^7^. For Micro: *N* = 8.0 × 10^6^, Mini: *N* = 2.7 × 10^6^, Small: *N* = 8.8 × 10^5^, Large: *N* = 1.6 × 10^5^. The empirical fits allow the computation of the number of locations in a certain range while choosing an arbitrary bin size. The number of potential pico hydropower locations would be larger if computations were performed on a finer-resolution grid. For the separation between the classes, a capacity factor of 0.5 was used.

We found that a log-log relationship exists between hydropower potential and the number of locations with that potential (Fig 3), though this relationship is not linear over the full range. It is interesting that this figure clearly resembles Fig 2 from ref [30], which depicted the volumetric capacity of artificial reservoirs as a function of the number of reservoirs that can be found globally. Apparently, reservoirs that have already been constructed, follow the same distribution as potential sites. The peak of the graph in the micro hydropower range is mainly caused by the limited resolution of our grid.

**Fig 4:**
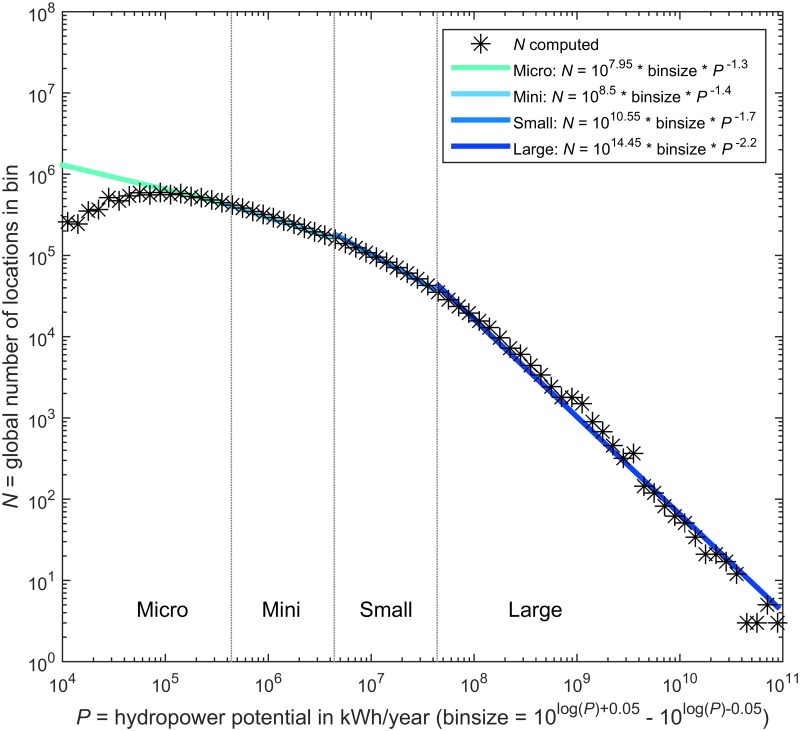
Global map of gross hydropower potential distribution. Large (>10MW), small (1–10 MW), mini (0.1–1 MW), micro (0.005–0.1 MW), and pico(<0.005MW) plants.

In addition to the number of locations, it is also important to estimate how much energy each of the different classes might theoretically be able to produce. We estimate that large hydropower plants deliver 68% of the total gross potential (Table 2), despite accounting for only 1.4% of all locations, small plants deliver 22% of the total, while the mini and micro provide a combined 10%. However, these mini and micro plants cover 91% of all locations and, compared with large plants, are more evenly distributed over the different continents (Fig 4).

**Table 2:** Hydropower potential per continent and its distribution among large, small, micro and mini plant sizes.

Plants:	Large (TWh/year)	Small (TWh/year)	Mini (TWh/year)	Micro (TWh/year)	Total (TWh/year)	
Asia	17,631	5,062	1,582	276	24,551	48%
North America	3,815	2,243	712	149	6,919	13%
Europe	971	854	328	86	2,240	4%
Africa	5,657	1,325	535	162	7,680	15%
South America	7,020	1,779	692	236	9,727	19%
Oceania	168	166	44	5	382	0.7%
Australia	34	84	46	14	177	0.3%
Global	35,296	11,513	3,939	929	51,677	100%
	68%	22%	8%	2%	100%	

Note that these numbers are the gross potential multiplied by a capacity factor of 0.5.

### 3.4 The hydropower database

Our analysis provides the community with two freely available products [31]: a raster file with all rivers and a point file with all potential hydropower locations. Each pixel of the raster file contains the average annual discharge. Quality of the products has been assessed through spot checks. Annual average discharge in the rivers compares well with the original runoff from the 30-minute dataset of the Global Runoff Data Centre [26]. Additionally, the location of the larger rivers derived from the GMTED2010 surface elevation data is accurate. This quality assessment is based on 100 spot checks in a graphical overlay of the simulated river network over satellite image data.

The point file with all potential hydropower locations contains 11.8 million locations with capacities between 8.76 MWh/year and 92 TWh/year. Approximately 4,800 locations show a potential capacity of more than 1 TWh/year, roughly 30% of which are located at existing hydropower plants. However, more exciting and promising are locations without an existing hydropower plant. For example, the Salween basin discharges 5,700 m^3^/s and has 29,600 locations with a gross potential of 981 TWh/year. Within the basin, 15 locations have a gross potential above 10 TWh/year with a total of 285 TWh/year. Myanmar, Thailand and China all have advanced plans to build over 20 dams in the Salween River Basin [32].

## 4 Discussion and conclusions

It has been shown that the systematic approach used here, based on DEM and discharge data, is suitable for calculating global hydropower potential. Our estimate of 52 PWh/year is somewhat more to the 41 PWh/year estimated in ref [11]. We showed that the estimate is sensitive to the vertical resolution of the DEM. Deteriorating the vertical resolution to 4 meters lowered our estimate to 29 PWh/year. New finer-resolution DEMs [33] and better discharge data will improve the estimates and reveal more locations in the pico hydropower scale.

Our database of potential hydropower [31], maps the locations where hydropower could be developed. However, for the development of hydropower sites, one also must consider the technical, economic and environmental factors [34,35]. These are subject to change over time, however ref [13] found the global ratio of technical, economic and exploitable to gross currently to be 20%, 16% and 13%.Whether such sites should be developed for electricity generation is also subject to political considerations [36]. Micro hydropower tends to be subject to more local decision making processes and can be vital for providing rural communities with access to renewable energy. As such, our database should be considered as the natural potential without our human considerations. Climate change is expected to slightly increase this potential [37].

Compared to the current global energy use of over 155 PWh/year [1,27], a gross potential of 52 PWh/year is considerable. Many developing countries listed in Table 2 have major undeveloped hydropower potential, and there is a big opportunity to develop hydropower combined with other economic activities such as irrigation [38]. Due to the existing trend of depleting oil and gas resources, and the desire to reduce CO2 emissions, we postulate that even locations that are currently not considered economically feasible, will, in the nearby future, expand hydropower production.
